# Assessment of a novel, capsid-modified adenovirus with an improved vascular gene transfer profile

**DOI:** 10.1186/1749-8090-8-183

**Published:** 2013-08-09

**Authors:** Katie M White, Raul Alba, Alan L Parker, Audrey F Wright, Angela C Bradshaw, Christian Delles, Robert A McDonald, Andrew H Baker

**Affiliations:** 1 BHF Glasgow Cardiovascular Research Centre, Institute of Cardiovascular and Medical Sciences, College of Medical, Veterinary and Life Sciences, University of Glasgow, 126 University Place, Glasgow G12 8TA, UK; 2 Present Address: Nanotherapix, S.L, Parc Empresarial Can Sant Joan, Avda. de la Generalitat 152-158, 08174, Sant Cugat del Vallès, Barcelona, Spain; 3 Present Address: Institute of Cancer and Genetics, Tenovus Building, Cardiff University, Heath Park, Cardiff CF14 4XN, Wales, UK

**Keywords:** Adenovirus, Vascular gene therapy, TIMP-3, Vein graft failure

## Abstract

**Background:**

Cardiovascular disorders, including coronary artery bypass graft failure and in-stent restenosis remain significant opportunities for the advancement of novel therapeutics that target neointimal hyperplasia, a characteristic of both pathologies. Gene therapy may provide a successful approach to improve the clinical outcome of these conditions, but would benefit from the development of more efficient vectors for vascular gene delivery. The aim of this study was to assess whether a novel genetically engineered Adenovirus could be utilised to produce enhanced levels of vascular gene expression.

**Methods:**

Vascular transduction capacity was assessed in primary human saphenous vein smooth muscle and endothelial cells using vectors expressing the LacZ reporter gene. The therapeutic capacity of the vectors was compared by measuring smooth muscle cell metabolic activity and migration following infection with vectors that over-express the candidate therapeutic gene tissue inhibitor of matrix metalloproteinase-3 (TIMP-3).

**Results:**

Compared to Adenovirus serotype 5 (Ad5), the novel vector Ad5T*F35++ demonstrated improved binding and transduction of human vascular cells. Ad5T*F35++ mediated expression of TIMP-3 reduced smooth muscle cell metabolic activity and migration *in vitro.* We also demonstrated that in human serum samples pre-existing neutralising antibodies to Ad5T*F35++ were less prevalent than Ad5 neutralising antibodies.

**Conclusions:**

We have developed a novel vector with improved vascular transduction and improved resistance to human serum neutralisation. This may provide a novel vector platform for human vascular gene transfer.

## Background

Pre-clinical studies indicate that gene delivery to the vasculature could potentially be utilised to treat a range of cardiovascular diseases such as vein graft failure [1], in-stent restenosis [2] and peripheral vascular disease [3]. Although clinical trials have generally identified no serious side effects, phase II and III randomised control trials have failed to achieve the promising efficacy seen in animal models and earlier trials [4-7], suggesting that further optimisation of the treatments is required. One of the more successful phase II clinical trials utilised intracoronary injection of an Adeno-associated Virus-1 vector to over-express the sarcoplasmic reticulum Ca^2+^-ATPase gene (*SERCA2a*) in patients with advanced heart failure [8]. This treatment significantly improved several cardiovascular parameters [8] and demonstrates the future potential of gene therapy treatments targeting the cardiovascular system. Critical to realising this potential is the development of more efficient gene delivery systems to generate sufficient levels of therapeutic transgene expression, specifically at the target site without causing any adverse effects.

In general, the commonly used viral vector Ad5 poorly transduces the vasculature, although, when high doses of virus are applied to intact blood vessels this vector can produce high levels of transgene expression in endothelial cells (EC) and, depending on the level of damage to the endothelial layer, lower levels in smooth muscle cells (SMC) [9-11]. Clearly, clinical indications define the route of vector delivery and this often requires short exposure times of virus to the vessel wall, limiting the level of gene delivery achievable. Analysis of the vascular expression of the coxsackie and adenovirus receptor (CAR), a primary Ad5 receptor, has shown that EC and SMC from a number of different vascular beds express a very low level of CAR [12-14], and analysis of intact human blood vessels could not detect CAR expression [15]. Havenga et al. have previously demonstrated that Ad5 vectors pseudotyped with the fiber from several subgroup B viruses show enhanced transduction of human vascular cells compared to Ad5 [16] and the Ad subgroup B receptor CD46 has been shown to be relatively highly expressed by vascular cells [12]. Therefore to maximise uptake into the vessel we have focused on developing a vector which utilises CD46 and not CAR as the principal receptor for binding to the cell surface.

An additional clinical consideration associated with the use of Ad5 vectors is that between 30 and 93% of the population (depending on the location of population) have pre-existing Ad5 neutralising antibodies [17,18], which can significantly limit the efficacy of transgene expression [19]. It has been demonstrated that modifying either the hexon protein [20] or pseudotyping Ad5 with the fiber of other Ad serotypes [21] can reduce the propensity of serum neutralisation of the virus.

Bio-distribution of the virus is also an important consideration when assessing potential off-target effects of gene therapy. Studies have shown that following catheter mediated delivery of an Ad5 vector to rabbit aortas, substantial transgene expression was detected in non-target tissues including hepatocytes, circulating monocytes and the testis [22], indicating that vector leakage from the target vascular tissue may remain a potential safety issue even when direct application of the vector to the vasculature is used. Hepatocyte uptake of Ad5 is mediated by the Ad5 hexon protein binding to circulating coagulation factor X (FX) [23,24], which then acts as a bridge to heparan sulphate proteoglycans [25]. Mutation of the FX binding site in the hexon protein has been shown to significantly reduce liver uptake [26-28]. Thus ablation of FX binding may offer the additional advantage of limiting any potential off-target effects.

In order to maximise gene delivery to vascular cells, whilst minimising the dose of virus required, we aimed to develop a modified Ad vector with the required attributes to produce enhanced vascular transduction. We have combined selective hexon amino acids mutations to ablate FX binding and simultaneous fiber pseudotyping in order to engineer a vector with improved gene delivery to vascular cells.

## Methods

### Ethics approval

Ethical approval (Ref 06/S0703/110) was obtained for the use of human saphenous vein and serum samples from patients undergoing coronary artery bypass grafting (CABG).

### Generation of Ad vectors

All viruses were based on the AdEasy, E1/E3-deleted Ad5 vector (Stratagene, Leicester, UK). Ad5 LacZ vectors were generated as previously described [26]. TIMP-3 expressing viruses were produced by cloning the TIMP-3 transgene into pShuttle-CMV plasmid (Stratagene). Homologous recombination between pShuttle-CMV TIMP-3 and the capsid encoding plasmids pAdEasy, pAd5CMV-HVR5*7*E451Q (Ad5T*) and pAd5CMV-HVR5*7*E451Q/F35++ (Ad5T*F35++) [26] was performed in BJ5183 electro-competent cells. Viruses were generated by transfection of HEK293 cells. All viruses were purified by CsCl gradient centrifugation and titred by end-point dilution to quantify plaque forming units (pfu) [29]. Particle titres were calculated from protein concentration using the Micro-Bicinchoninic Acid Protein Assay kit (Thermo Scientific, Cramlington, UK) and the formula 1 μg protein = 4x10^9^ virus particles [29] and confirmed by NanoSight measurement (NanoSight, Wiltshire, UK).

### Cell culture

All cells were cultured in a humidified atmosphere with 5% CO_2_ at 37°C. HSVSMC were isolated from medial explants of human saphenous vein samples received from patients undergoing CABG surgery. Cells were cultured in Smooth Muscle Cell Growth Medium 2 (PromoCell, Heidelberg, Germany) supplemented with 15% foetal bovine serum (PAA Laboratories, Somerset, UK), 2 mM L-Glutamine (Invitrogen, Paisley, UK) and 1% penicillin-streptomycin (Invitrogen). HSVEC were obtained by enzymatic collagenase digestion of human saphenous veins and were maintained in Large Vessel Endothelial Cell Basal Medium (TCS CellWorks, Buckinhgham, UK) supplemented with 20% foetal bovine serum (PAA Laboratories), 2 mM L-Glutamine (Invitrogen) and 1% penicillin-streptomycin (Invitrogen, Paisley, UK). A549 cells (human lung carcinoma, ATCC CCL-185) were maintained in RPMI160 supplemented with 10% foetal bovine serum (PAA Laboratories), 2 mM L-Glutamine (Invitrogen) and 1% penicillin-streptomycin (Invitrogen).

### Analysis of virus:cell binding

Cells were seeded at 2x10^5^ cells/well in 24 well plates. The following day cells were cooled to 4°C for 30 minutes, washed in PBS then incubated for 1 hour at 4°C in serum free media containing 5000 vp/cell of virus. DNA was extracted using the QIAmp DNA Mini Kit (Qiagen, West Sussex, UK) according to the manufacturer’s protocol. DNA was quantified using ND-1000 Nanodrop spectrophotometer. Viral genomes were quantified in 100 ng DNA by quantitative SyBR green real time PCR (Applied Biosystems, Cheshire, UK) using 0.2 μM hexon specific primers [30].

### *In vitro* transduction of vascular cells

Cells were seeded at 2x10^4^ cells/well in 96 well plates. The following day cells were infected with 5000 vp/cell in serum free media. Cells were incubated for 3 hours at 37°C, washed in PBS then incubated for a further 48 hours in complete media.

For antibody blocking experiments cells were pre-incubated for 1 hour at 4°C in serum free media containing 5 μg/ml mouse anti human CD46 antibody MEM-258 (AbD Serotec, Oxford, UK) or mouse IgG1 isotype control (Dako, Glostrup, Denmark). Infections and transgene quantification were then performed as described above.

### β-galactosidase detection

For visualisation of β-Galactosidase expression, cells were washed in PBS, fixed in 4% PFA and stained in X-gal. β-Galactosidase activity was quantified using Tropix Galacton Plus (Applied Biosystems) according to the manufacturer’s instructions. Protein concentrations were measured by BCA assay (ThermoScientific, Leicestershire, UK) according to the manufacturer’s instructions. Absorbances were measured using a Wallac VICTOR2 (PerkinElmer Life and Analytical Sciences, Boston, USA) and values were expressed as relative light units/mg protein.

### Serum neutralisation assay

Human serum samples (102 patients) were obtained from a Scottish cohort of patients undergoing CABG surgery. Based on a previously established protocols [21,31] A549 cells were infected with 10000 vp/cell in the presence or absence of 2.5% serum. β-Gal activity was measured 48 hours post infection and normalised to total protein levels as described above. Serum samples which caused >90% inhibition of transduction were considered to be neutralising.

### Detection of TIMP-3 expression

Infections were performed as described above. 36 hours post infection 1:1000 dilution of Monensin (Biolegend, London, UK) was added to the culture medium and incubated for a further 12 hours.

For immunocytochemistry, cells were permeabilised in PBS-Tween, blocked in goat serum (Dako), then incubated with Rabbit anti-human TIMP-3 antibody (Millipore) or Rabbit IgG control. Goat anti-rabbit Alexa 546 (Invitrogen) was used for detection. Slides were mounted using Prolong Gold with DAPI (Invitrogen) and imaged using Zeiss confocal imaging system LSM500.

For Western blotting, denatured cell samples were separated on a 12% SDS polyacrylamide gel. TIMP-3 expression was detected using Rabbit anti-human TIMP3 antibody (Millipore), and swine anti-rabbit-HRP antibody (Dako). Blots were stripped and re-probed using mouse anti-human β-actin monoclonal antibody (AbCam) and rabbit anti-mouse-HRP (Dako).

### Scratch assay

Cells were infected as described above. A previously described scratch assay was then used [32]. Briefly, 48 hours post infection, a 200 μl pipette tip was used to produce three evenly sized vertical scratches per well, cells were washed with PBS and placed in fresh media. Images were captured at 0, 12 and 19 hours post-scratch.

### Cell metabolic activity

Cells were infected as described above. Metabolic activity was analysed 48 hours post infection using the CellTiter96® AQ_ueous_ One Solution Cell Proliferation Assay (Promega).

### Statistics

Results presented are representative data from a minimum of three separate experiments with three experimental replicates per group. All results are shown as mean+/−SEM. Values were compared using One-way-ANOVA with Tukey’s Multiple Comparison Test. p < 0.05 was considered significant.

## Results and discussion

### *In vitro* binding and transduction profiles of a CD46 binding Ad vector in human vascular cells

Surface plasmon resonance has previously demonstrated that mutations in the hexon protein of the virus Ad5T* prevent binding to FX, and following systemic delivery in mice, this ablation of FX binding causes a significant reduction in liver tropism [26]. Pseudotyping this vector with the Ad35++ fiber (Ad5T*F35++) enables cellular binding and transduction to occur through an interaction with CD46 [26]. To exploit the relatively high level of CD46 expression on human vascular cells [12] we investigated the efficacy of Ad5T*F35++ for gene delivery to these cells.

**Figure 1:**
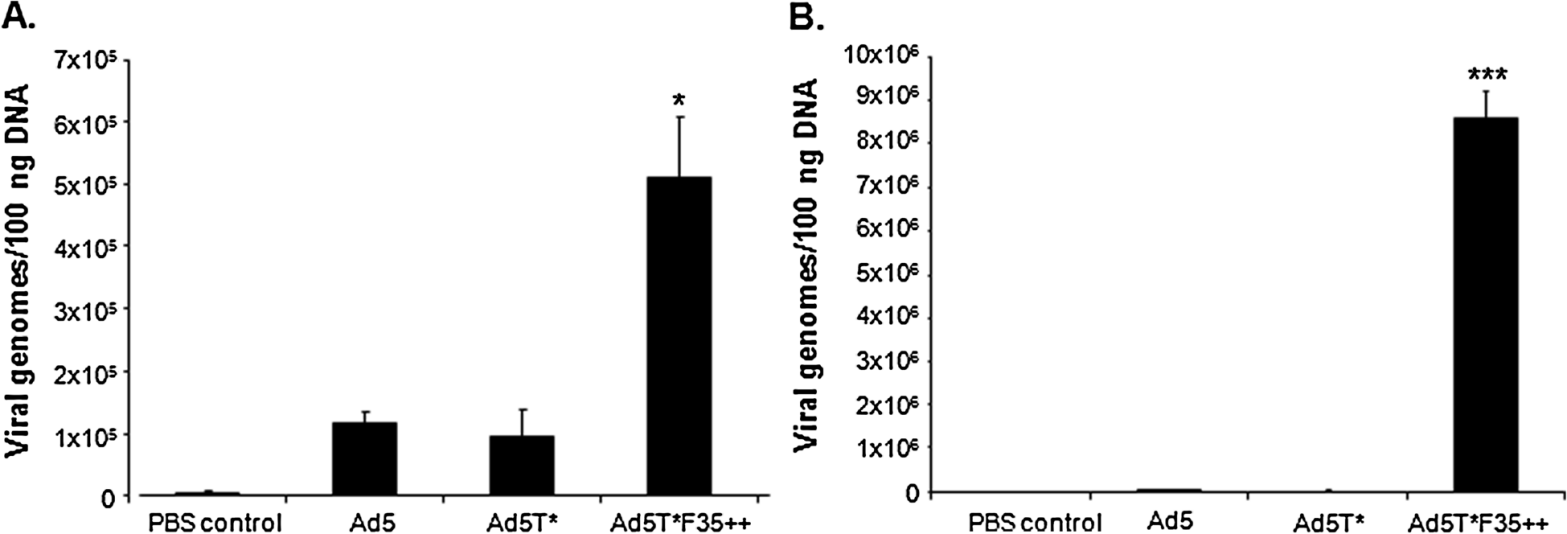
***In vitro *profiling of virus binding to A. HSVSMC and B. HSVEC.** 5000 vp/cell were allowed to bind cells for 1 hour at 4°C. Ad genomes were detected by quantitative PCR (* = p < 0.05 vs Ad5, *** = p < 0.001 vs Ad5).

**Figure 2:**
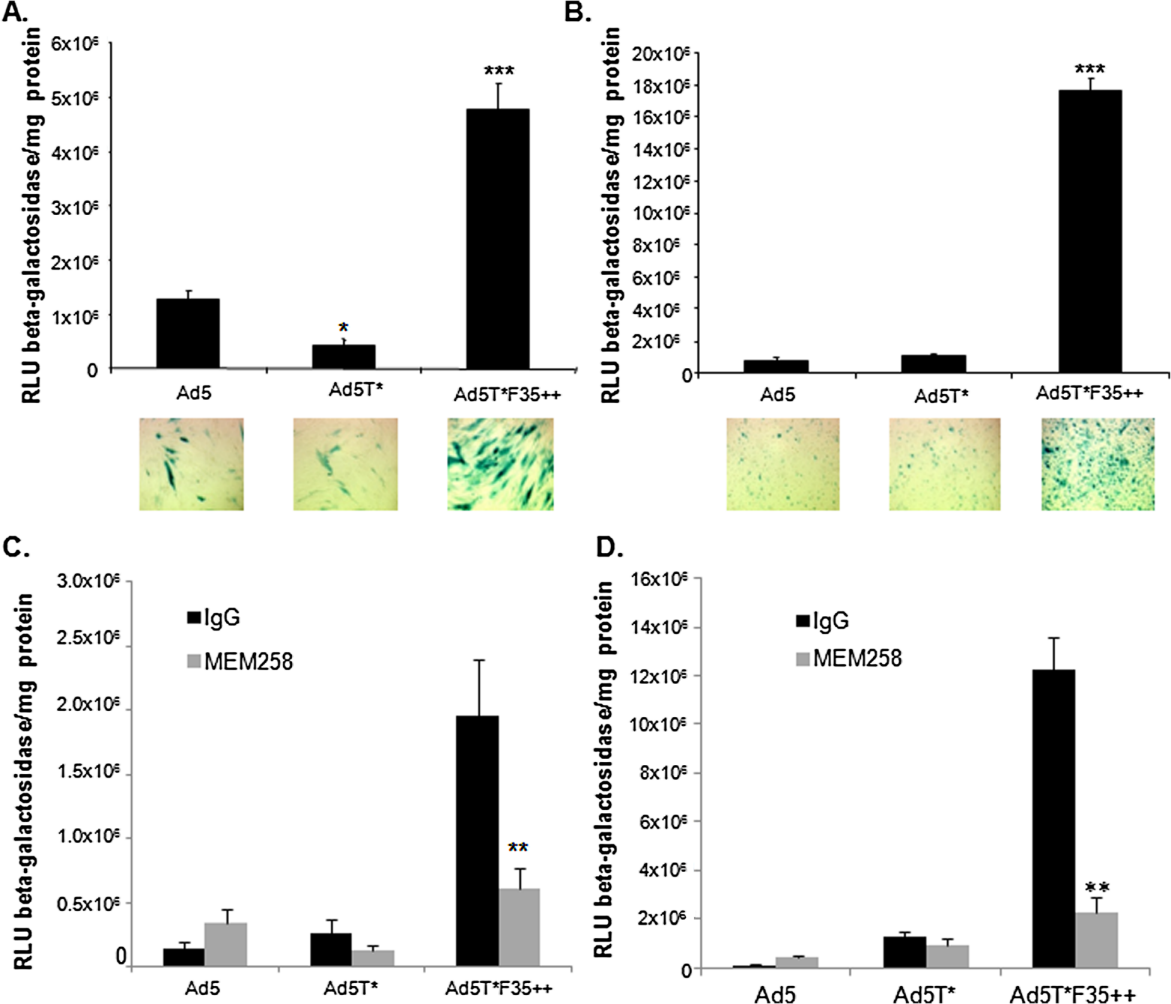
***In vitro *virus transduction.** β-Gal activity and representative images of X-gal stained cells in **A.** HSVSMC and **B.** HSVEC infected with 5000 vp/cell for 3 hours at 37°C. β-Gal activity was normalised to total protein levels (* = p < 0.05 compared to Ad5, *** = p < 0.001 compared to Ad5). β-Gal activity normalised to total protein in **(C)** HSVSMC and **(D)** HSVEC infected with 5000 vp/cell in the presence or absence of CD46 function-blocking antibody MEM258 or an isotype matched control. (* = p < 0.05 relative to isotype control, ** = p < 0.01 relative to isotype control).

Initially we performed *in vitro* cell binding and transduction experiments with β-galactosidase expressing vectors to compare Ad5, Ad5T* and Ad5T*F35++. Ad5T*F35++ showed significantly higher levels of binding to human saphenous vein smooth muscle cells (HSVSMC) and human saphenous vein endothelial cells (HSVEC) compared to Ad5 (Figure 1). This correlated with increased cellular transduction as Ad5T*F35++ produced significantly higher levels of transgene expression in both HSVSMC (Figure 2A) and HSVEC (Figure 2B).

To confirm that Ad5T*F35++ utilises CD46 as a receptor, virus transduction was measured in the presence of the CD46 specific antibody MEM258. The transduction of both HSVSMC (Figure 2C) and HSVEC (Figure 2D) was significantly reduced by the presence of MEM258, confirming that the virus interaction with CD46 is a critical step in the transduction pathway using this modified virus.

Therefore Ad5T*F35++ was found to bind to and transduce HSVEC and HSVSMC more efficiently than Ad5, and so may provide a more effective vector for gene delivery to the vasculature.

### Ad5T*F35++ expression of TIMP-3 inhibits SMC migration and proliferation

To demonstrate the potential therapeutic use of Ad5T*F35++, we produced a vector with this modified configuration and an expression cassette encoding tissue inhibitor of matrix metalloproteinase 3 (TIMP-3). TIMP-3 is a promising candidate gene for gene therapy to prevent vein graft failure as it has been shown to inhibit matrix metalloproteinase activity and it promotes vascular SMC apoptosis [1,33,34]. It has previously been demonstrated that adenoviral mediated over-expression of TIMP-3 in porcine vein grafts significantly reduces neointima formation in both a short term model [33] and up to 3 months post engraftment [1].

**Figure 3:**
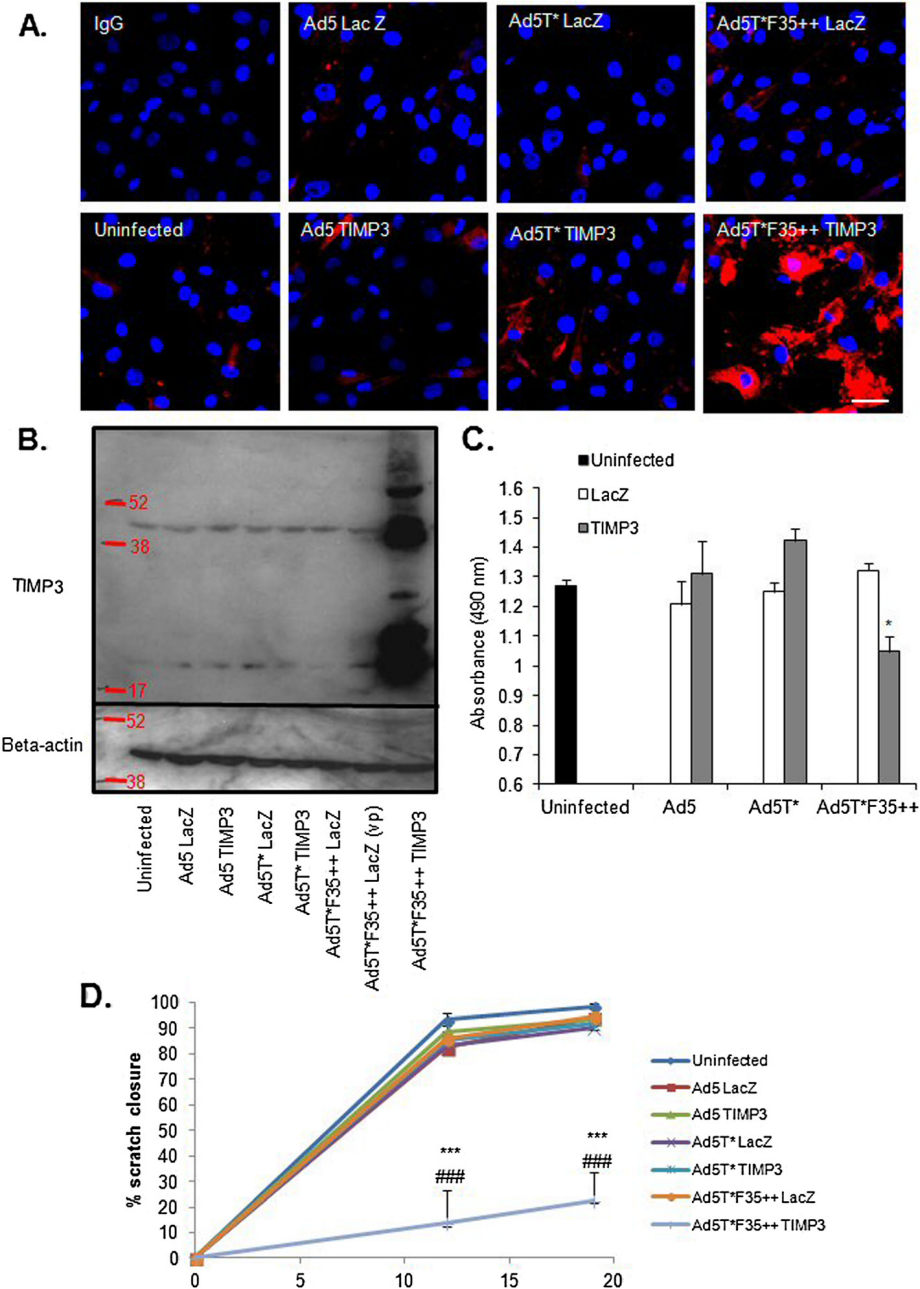
**Effect of TIMP-3 over-expression in HSVSMC.** HSVSMC were infected with 5 pfu/cell TIMP-3 expressing viruses. **A.** Representative images of immunocytochemistry performed using rabbit anti-human TIMP-3 antibody (red) or the appropriate IgG control (in Ad5T*F35++ TIMP-3 infected cells). Scale bar = 10 μm **B.** Western blot to detect TIMP-3 expression, with equivalent protein loading confirmed by β-actin detection. Ad5T*F35++ LacZ infections were performed with both an equivalent pfu/cell and vp/cell as Ad5T*F35++ TIMP-3. **C.** MTS assay performed 48 hours post infection. *p < 0.05 vs equivalent LacZ virus. **D.** Scratch assays were performed 48 hours post infection. The scratch width was measured at 0, 12 and 19 hours and the percentage scratch closure was calculated. ***p < 0.001 vs LacZ and ###p < 0.001 vs uninfected.

In order to investigate the potential benefit of the enhanced efficiency of the Ad5T*F35++ vector, experiments using the TIMP-3 expressing vectors were performed using a low dose of virus (5 pfu/cell). TIMP-3 over-expression in transduced HSVSMC was confirmed by immunocytochemistry (Figure 3A) and western blots (Figure 3B). This demonstrated that Ad5T*F35++ TIMP-3 transduced cells with high efficiency and mediated high levels of TIMP-3 expression, whereas both control vectors, Ad5 TIMP-3 and Ad5T* TIMP-3, led to the production of low levels of ectopic TIMP-3.

To determine if the level of TIMP-3 expression achieved was sufficient to have a functional effect, metabolic assays were performed on transduced cells. In cells transduced with Ad5T*F35++ TIMP-3, cell metabolism was significantly reduced compared to Ad5T*F35++ LacZ (Figure 3C). Infection of the Ad5 and Ad5T* vectors caused no significant reduction in metabolic activity (Figure 3C). As TIMP-3 has also previously been shown to reduce SMC migration, wound healing assays were performed in transduced cells. Only Ad5T*F35++ TIMP-3 was found to significantly reduce migration compared to the equivalent LacZ control virus (Figure 3D). This suggests that due to the more efficient vascular transduction of Ad5T*F35++, compared to Ad5, a lower dose of vector may potentially be required to mediate a therapeutic effect, although this remains to be fully tested.

Earlier studies in this field suggested that the use of Ad vectors to deliver genes to the vasculature can cause an increase in neointima formation, probably due to a detrimental inflammatory response to the vector [35]. Although this has not been seen in subsequent pig models [1], the inflammatory response to any vector needs to be carefully investigated when considering clinical use. The increased transduction efficiency of the Ad5T*F35++ may enable the use of a lower dose to achieve a therapeutic effect and therefore may be less likely to provoke an inflammatory response. Also, a recent study has indicated that the innate immune response to Ad is at least in part initiated by binding to FX [36], therefore ablating FX binding may also reduce the innate immune response that acts against the vector.

### Serum neutralisation of virus transduction

**Figure 4:**
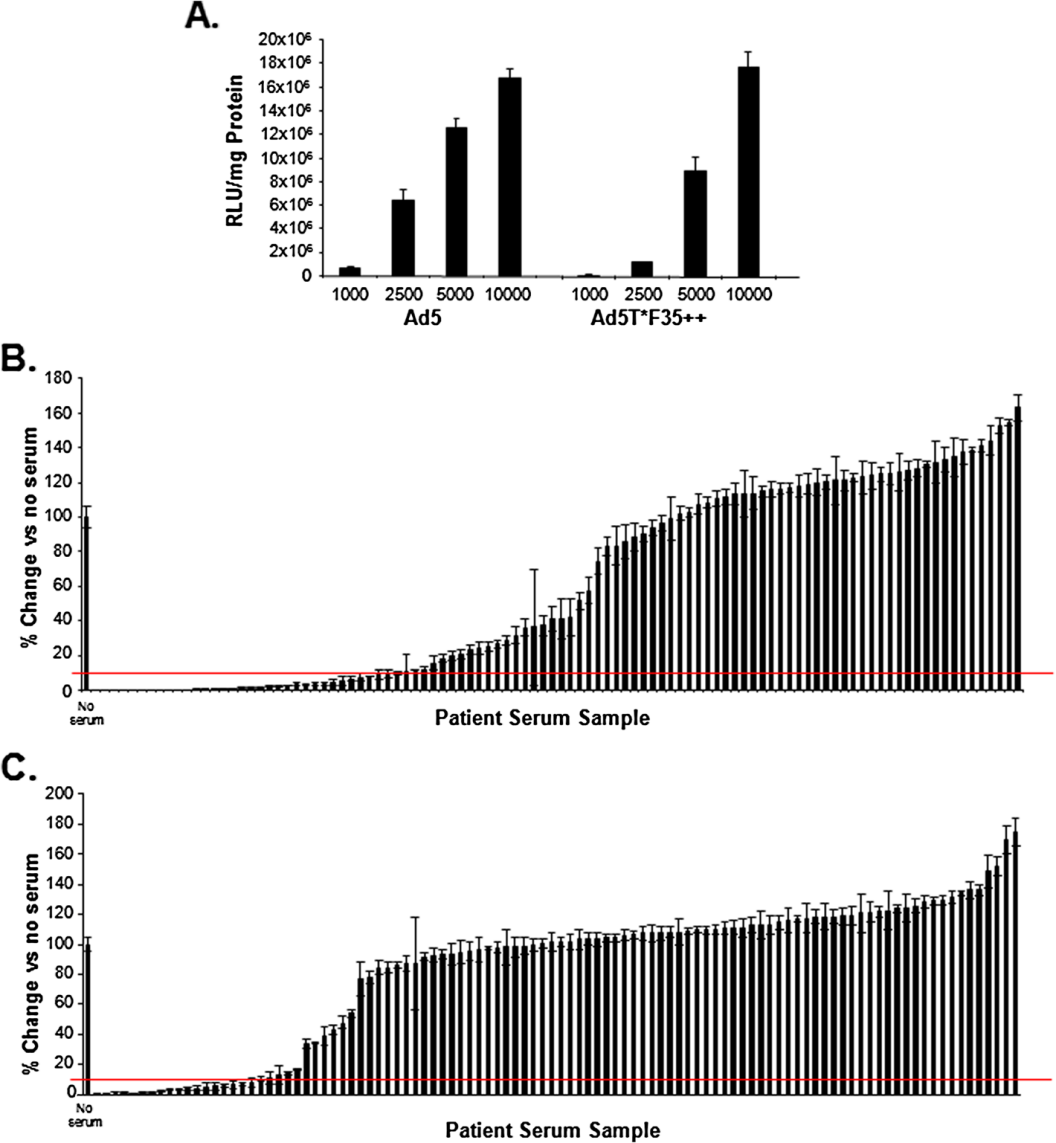
**Effect of human sera on virus transduction. A.** A549 cells were infected with a range of vp/cell as indicated. β-Gal activity was normalised to total protein levels. **B-C.** A549 cells were infected with 10,000 vp/cell **B.** Ad5, **C.** Ad5T*F35++ in the presence of 2.5% human sera. β-Gal activity was measured 48 hours post infection and normalised to total protein levels. Graphs show the relative change in transduction compared to no serum control. The red line indicates 90% inhibition of virus transduction compared to no serum control sample.

Another important consideration when developing a vector for clinical applications is the effect of pre-existing neutralising antibodies. Previous studies have provided evidence of neutralising antibodies against both Ad5 hexon [18] and fiber proteins [21] which significantly reduce virus transduction [37]. Incorporation of hexon mutations [20] or fiber pseudotyping [21] has been demonstrated to reduce the neutralisation of Ad5 based vectors. In addition to improving vascular transduction through altering receptor usage, the Ad5T*35++ capsid modifications may also affect the ability of pre-existing antibodies in human serum to neutralise the virus. To determine if this is the case, transduction assays were performed in the presence of a panel of 102 human serum samples. The assay was performed in A549 cells as transduction of the viruses was found to be comparable in this cell type (Figure 4A). Thirty-three percent of serum samples were found to reduce Ad5 transduction by at least 90%, (Figure 4B) where as Ad5T*F35++ transduction (Figure 4C) was neutralised by 18% of serum samples. Therefore, we have shown that through modifying the virus hexon and fiber protein we have engineered a vector which is less likely to be neutralised by a patient’s pre-existing antibodies.

## Conclusions

We have demonstrated that by combining mutations of the Ad5 hexon and pseudotyping with the Ad35++ fiber we have developed a vector which has many attributes that suggest it could be a promising vector for efficient gene delivery to the vasculature for the treatment of cardiovascular diseases such as vein graft failure and in-stent restenois. Further testing of this vector using *in vivo* models is required but, due to differences in CD46 expression patterns in rodents [38] and non-human primates [39] and the low level of homology between porcine and human CD46 [16], there are no appropriate animal models to further test the efficacy of this vector. However, the efficacy of the virus can be further tested using an established *ex vivo* model of neointima formation in cultured human veins [40].

## Abbreviations

Ad5: Adenovirus serotype 5; CABG: Coronary artery bypass grafting; CAR: Coxsackie and adenovirus receptor; EC: Endothelial cells; FX: Factor X; HSVEC: Human saphenous vein endothelial cells; HSVSMC: Human saphenous vein smooth muscle cells; Pfu: Plaque forming ubits; SMC: Smooth muscle cells; TIMP-3: Tissue inhibitor of matrix metalloproteinase-3; Vp: Virus particles.

## Competing interests

The authors declare that they have no competing interests.

## Authors’ contributions

KMW, RA and ACB produced the vectors. AFW and RA carried out the cell binding and transduction assays with the reporter gene vectors. ALP preformed the serum neutralisation assays. CD obtained ethical approval and provided the human samples. KMW performed all experiments with the TIMP-3 expressing vectors and drafted the manuscript. RAM and AHB participated in the study conception and design. All authors read and approved the final manuscript.
